# Age-dependent associations of sleep duration and insomnia symptoms with cognitive decline across five decades of adult life: a multi-cohort longitudinal study

**DOI:** 10.64898/2026.09.25.26363056

**Published:** 2026-09-28

**Authors:** Yi Fang, Allysa Quick, Nina Oryshkewych, Nicole Fernandez, Andrew S Lim, Lisa L Barnes, David A Bennett, Andrew Steptoe, Rebecca Hardy, Séverine Sabia, Meredith L Wallace, Yue Leng

## Abstract

**Background:**

Associations of sleep duration and insomnia symptoms with cognitive decline are inconsistent, and whether they represent modifiable risk factors or early manifestations of neurodegeneration may depend on life stage. We examined how sleep-cognition associations vary with age (44 to >90 years).

**Methods:**

We studied 16,884 participants from three cohorts with harmonised measures. In the English Longitudinal Study of Ageing (ELSA: n=9,473, median age 64, range 50 to ≥90), linear mixed-effects models examined age-specific differences in cognitive change (recall and fluency) associated with sleep duration, difficulty falling asleep, nocturnal awakenings, incorporating sleep-by-age-by-time interactions, with initial adjustment for sociodemographic factors and further adjustment for health and lifestyle factors. Sleep characteristics showing consistent age-dependent associations in ELSA were further evaluated in Whitehall II (n=5,580, median age 55, range 44 to 68) and the Rush Memory and Aging Project (MAP: n=1,831, median age 80, range 53 to 102).

**Findings:**

In ELSA, difficulty falling asleep ≥1 time/week (vs <1 time/week) was associated with less favourable 5-year change in recall at age 50 (difference −0·12 SD, 95% CI −0·20 to −0·04), with the association attenuating and reversing towards more favourable change at older ages (p-interaction=0·009 [linear], 0·016 [quadratic]). These persisted after further adjustment for health and lifestyle factors. Short sleep (≤6 h) showed similar patterns that attenuated after further adjustment. Similar age gradients were observed for difficulty falling asleep in Whitehall II and MAP. There was no evidence of age modification on long sleep and nocturnal awakenings.

**Interpretation:**

The cognitive implications of sleep disturbances vary by life stage. In midlife, difficulty falling asleep was associated with faster cognitive decline, supporting its role as a modifiable risk factor. In advanced age, the reversal is consistent with diminished arousal capacity accompanying prodromal dementia.

**Funding:**

US National Institute on Aging

**Research in context:**

**Evidence before this study**

We searched PubMed and Web of Science from database inception to Jan 1, 2026, using terms related to sleep, sleep duration, insomnia, cognitive decline, dementia, and ageing, without language restrictions. Findings on sleep and cognitive ageing are inconsistent and vary by population studied. In the few prospective studies of midlife populations, short sleep and insomnia symptoms have been linked to increased dementia risk, but evidence remains limited. In older populations, findings are more mixed, with several studies reporting null associations and others reporting counterintuitive associations between greater sleep complaints and better cognitive outcomes. Few studies span a sufficient age range to formally test age as an effect modifier; those that did were often limited by modest sample sizes, binary age stratification, or failure to model time within the interaction.

**Added value of this study**

To our knowledge, this is the first multi-cohort study to examine age as a modifier of the association between sleep and longitudinal cognitive trajectories across general population spanning midlife to the oldest old age. In a nationally representative UK cohort, we show that difficulty falling asleep has robust age-dependent associations with cognitive decline: faster decline in midlife, attenuation through later adulthood, and reversal in the oldest old. Short sleep duration shows a similar but less robust age-dependent pattern, partly explained by health-related and lifestyle factors. These findings were replicated across two independent cohorts in the UK and US with complementary age ranges.

**Implications of all the available evidence**

The available evidence supports age-specific interpretation of sleep disturbances in relation to cognitive health. In midlife, difficulty falling asleep is associated with faster cognitive decline, supporting its potential as a modifiable target for intervention. In the oldest old, difficulty falling asleep may reflect lower sleep propensity from preserved wake-promoting function, consistent with converging evidence linking increased sleepiness to neurodegeneration.

## Full Text Availability

The license terms selected by the author(s) for this preprint version do not permit archiving in PMC. The full text is available from the preprint server.

